# Language switching ability and executive function development in English learners: a longitudinal investigation of bilingual cognitive control

**DOI:** 10.1186/s40359-025-03747-0

**Published:** 2025-11-28

**Authors:** Shuai Wang

**Affiliations:** College of Fundamental Education, Shanghai Civil Aviation College, Shanghai, 200235 China

**Keywords:** Bilingual cognitive control, Language switching, Executive function, English learners, Longitudinal development, Inhibitory control

## Abstract

This longitudinal study examines the developmental relationships between language switching ability and executive function components in English learners across multiple proficiency levels. Drawing on bilingual cognitive control theory, we investigated how working memory, inhibitory control, and cognitive flexibility interact with language switching performance over an 18-month period. Participants (*N* = 266) completed comprehensive assessments of language switching tasks, executive function measures, and English proficiency tests at six time points. Results revealed systematic improvements in both language switching efficiency and executive function performance, with switching costs and mixing costs showing significant reductions over time. Growth curve modeling demonstrated bidirectional relationships between executive function components and language switching ability, with inhibitory control and cognitive flexibility emerging as stronger predictors than working memory. Individual differences in developmental trajectories were significantly moderated by English proficiency level, age, and baseline executive function capabilities. Intermediate proficiency learners exhibited the steepest improvement curves, suggesting an optimal developmental window for cognitive control enhancement. These findings provide empirical support for theoretical models proposing that language switching practice strengthens domain-general executive control systems while executive function capabilities facilitate more efficient bilingual language management.

## Introduction

### Theoretical foundation and research significance

Bilingual cognitive control theory has emerged as a pivotal framework for understanding the complex mechanisms underlying language processing and cognitive flexibility in multilingual individuals [1]. This theoretical perspective posits that bilingual speakers develop enhanced executive control systems through the constant management of competing linguistic systems, resulting in cognitive advantages that extend beyond language-specific domains [2]. The significance of this theory lies in its capacity to explain how individuals navigate between different linguistic codes while maintaining communicative efficiency and cognitive coherence.

English learners, representing a substantial population of emerging bilinguals worldwide, provide a unique lens through which to examine the development of cognitive control mechanisms [3]. The process of acquiring English as a second language involves continuous language switching and inhibitory control processes that fundamentally reshape cognitive architecture. Understanding these mechanisms is crucial for both theoretical advancement in cognitive science and practical applications in educational contexts.

### Current research landscape and existing limitations

Contemporary research on language switching abilities among English learners has primarily focused on behavioral manifestations and processing speed measures, while overlooking the underlying cognitive control mechanisms that facilitate these abilities [4]. Previous studies have predominantly examined language switching through reaction time paradigms and error rate analyses, providing limited insight into the dynamic relationship between executive functions and bilingual language control.

Significant gaps exist in our understanding of how executive function components specifically contribute to language switching proficiency in different stages of English acquisition [5]. Most existing research has adopted cross-sectional approaches that fail to capture the developmental trajectory of these interconnected cognitive systems. Furthermore, there is insufficient attention to individual differences in executive function development and their impact on language switching outcomes.

The majority of studies have concentrated on balanced bilinguals or heritage speakers, leaving the specific challenges and advantages of formal English learners relatively underexplored [6]. This limitation restricts our ability to generalize findings to the broader population of English language learners who acquire their second language through instructional contexts rather than natural immersion environments.

### Executive function and Language switching interface

Executive function encompasses three core components: inhibitory control, working memory, and cognitive flexibility, each playing distinct yet interconnected roles in bilingual language processing [7]. Inhibitory control enables speakers to suppress irrelevant linguistic information and prevent cross-linguistic interference, while working memory maintains and manipulates linguistic representations during real-time language switching tasks. Cognitive flexibility facilitates the seamless transition between different linguistic rule systems and cultural communication patterns.

The relationship between these executive function components and language switching ability appears to be bidirectional, with language switching experience potentially strengthening executive control systems while robust executive functions enhance switching proficiency. This dynamic interaction suggests that English learners may develop specialized cognitive control mechanisms through their language learning experience, leading to measurable improvements in both linguistic and non-linguistic executive function tasks.

Research evidence indicates that the demands of language switching create a unique cognitive training environment that may accelerate executive function development beyond what is observed in monolingual populations. However, the specific developmental patterns and individual variation in these relationships remain poorly understood, particularly across different proficiency levels and learning contexts.

### Research questions and hypotheses

This study addresses three primary research questions: First, what are the developmental trajectories of language switching ability and executive function among English learners across different proficiency levels? Second, how do individual differences in executive function components predict language switching performance in various linguistic contexts? Third, what are the bidirectional influences between language switching experience and executive function development over time?

Based on bilingual cognitive control theory and existing empirical evidence, we hypothesize that English learners will demonstrate progressive improvements in both language switching efficiency and executive function performance as proficiency increases. We further predict that inhibitory control and cognitive flexibility will serve as stronger predictors of language switching ability than working memory, particularly in contexts requiring rapid linguistic code alternation. Additionally, we hypothesize that intensive language switching practice will contribute to enhanced executive function performance beyond language-specific improvements.

### Research innovation and contributions

This investigation introduces several methodological and theoretical innovations to the field of bilingual cognitive research [8]. First, it employs a longitudinal design that captures the dynamic development of cognitive-linguistic relationships over extended periods, addressing the limitations of cross-sectional approaches. Second, it integrates multiple assessment modalities including behavioral measures, cognitive tasks, and linguistic performance indicators to provide comprehensive evaluation of executive function-language switching interactions.

The study contributes to theoretical advancement by testing specific predictions derived from bilingual cognitive control theory within the context of formal language learning environments. Practically, the findings will inform evidence-based approaches to English language instruction that capitalize on the cognitive benefits of strategic language switching practice. The research addresses critical gaps in our understanding of how cognitive control mechanisms develop in response to second language learning challenges, providing insights relevant to both cognitive science and applied linguistics domains.

## Literature review and theoretical foundation

### Developmental trajectory of bilingual cognitive control theory

The theoretical foundation of bilingual cognitive control has evolved substantially since the early conceptualizations of bilingual language processing in the 1990s. Initial models primarily focused on language-specific processing mechanisms, treating bilingual cognition as the simple coexistence of two separate linguistic systems [9]. This perspective gradually shifted toward recognizing the dynamic interaction between languages and the cognitive control systems that manage linguistic competition.

Green’s inhibitory control model represents a seminal contribution to understanding bilingual language management, proposing that successful bilingual communication requires active inhibition of the non-target language to prevent interference [10]. This model introduced the concept of language schemas as task-specific control structures that regulate language selection through inhibitory mechanisms. The model suggests that speakers must suppress competing linguistic representations while simultaneously activating appropriate language-specific resources, creating a complex cognitive control challenge that demands sophisticated executive function capabilities. However, recent research has challenged certain aspects of this framework. While Green’s model has been influential in explaining language switching phenomena, critics argue that evidence for domain-general inhibitory mechanisms in language control remains inconsistent across studies, with some investigations failing to find the predicted inhibitory effects in balanced bilinguals (Costa & Santesteban, [11]; Declerck & Philipp, [12]). Furthermore, the model primarily addresses reactive control during language switching but provides limited explanation for proactive control mechanisms that bilinguals employ in anticipation of language alternation. These limitations have motivated theoretical developments toward more nuanced frameworks that acknowledge the heterogeneity of bilingual experiences and control strategies.

The inhibitory control framework has been instrumental in explaining phenomena such as language switching costs and cross-linguistic interference effects. Green’s model posits that language switching involves the sequential inhibition and activation of competing language schemas, with the degree of inhibition required varying based on the relative strength and accessibility of each language system. This theoretical perspective provides a mechanistic account of how bilinguals maintain communicative effectiveness despite managing multiple linguistic codes simultaneously.

Abutalebi’s neurocognitive model expanded upon Green’s theoretical foundation by incorporating neurobiological evidence and identifying specific brain networks involved in bilingual language control [13]. This model emphasizes the role of the anterior cingulate cortex, prefrontal cortex, and caudate nucleus in managing language selection and inhibitory control processes. Abutalebi’s framework demonstrates how cognitive control mechanisms are instantiated in neural architecture, providing empirical support for the theoretical predictions derived from behavioral models.

The neurocognitive approach has suggested that bilingual language control may engage domain-general executive function networks in addition to language-specific neural circuits [14]. However, the extent to which these networks overlap with general executive control systems remains a topic of ongoing investigation, with some studies reporting substantial overlap while others find more circumscribed correspondence. Recent neuroimaging evidence indicates that the relationship between bilingual language control and domain-general executive functions varies depending on individual differences in bilingual experience, including age of acquisition, language proficiency balance, and frequency of language switching (DeLuca, Rothman, Bialystok, & Pliatsikas, [15]). This finding suggests that the cognitive advantages observed in bilingual populations may result from the strengthening of both language-specific and general-purpose control systems through linguistic experience, with the relative contribution of each varying across different bilingual populations. The model particularly emphasizes the role of conflict monitoring and cognitive flexibility in successful language switching performance.

Contemporary theoretical developments have integrated these foundational models into more comprehensive frameworks that account for individual differences, contextual factors, and developmental changes in bilingual cognitive control [16]. Recent theoretical advances recognize that cognitive control mechanisms in bilingual processing are not static but rather adapt dynamically to changing linguistic demands and environmental contexts.

The adaptive control hypothesis represents a significant theoretical evolution, proposing that bilingual cognitive control systems adjust their operational characteristics based on the specific demands of different interactional contexts [17]. This framework suggests that the same individual may employ different control strategies depending on whether they are engaged in single-language contexts, dual-language contexts, or dense code-switching environments. The adaptive nature of bilingual cognitive control provides a more nuanced understanding of how executive function systems respond to varying linguistic challenges and explains the considerable individual differences observed in bilingual language switching abilities.

These theoretical developments have established bilingual cognitive control as a dynamic, adaptive system that extends beyond simple language management to encompass broader cognitive advantages and enhanced executive function capabilities.

### Cognitive mechanisms of Language switching ability

Language switching ability encompasses the cognitive capacity to alternate between different linguistic codes while maintaining communicative effectiveness and minimizing processing interference [18]. This multifaceted construct involves both voluntary and involuntary transitions between languages, requiring sophisticated coordination of inhibitory control, working memory, and cognitive flexibility systems. Recent theoretical frameworks (e.g., Green & Abutalebi, [17]; Declerck & Philipp, [12]) emphasize the dynamic nature of language switching, reconceptualizing it as an active cognitive process involving anticipatory preparation, reactive control, and sustained monitoring rather than a passive alternation between linguistic systems. This conceptualization recognizes that language switching demands continuous regulation of linguistic competition through multiple control mechanisms operating at different processing stages, from conceptual activation through lexical selection to articulatory planning.

Measurement approaches for assessing language switching ability have predominantly relied on experimental paradigms that quantify behavioral performance through reaction time analysis and error rate assessment [19]. The most widely employed methodology involves language switching tasks where participants name stimuli or respond to cues while alternating between languages according to predetermined sequences or external signals. These paradigms typically measure both voluntary switching, where participants control the timing and direction of language changes, and cued switching, where external stimuli determine language selection requirements.

Switching costs represent a fundamental metric in language switching research, reflecting the additional cognitive effort required when transitioning from one language to another compared to maintaining the same language [20]. These costs manifest as increased reaction times and elevated error rates on switch trials relative to non-switch trials, providing quantifiable evidence of the cognitive demands associated with language alternation. Research has consistently demonstrated that switching costs vary systematically based on factors such as language proficiency, switching direction, and contextual demands.

The asymmetrical nature of switching costs has emerged as a particularly important finding, with switches from the weaker to stronger language typically producing smaller costs than switches in the opposite direction [21]. This asymmetry suggests that different cognitive mechanisms may be engaged depending on the relative accessibility and inhibitory demands of the target and non-target languages. The pattern indicates that suppressing a dominant language while accessing a weaker language requires greater cognitive control resources than the reverse transition.

Mixing costs constitute another core concept in language switching research, representing the performance decrement observed when languages are intermixed compared to monolingual contexts, even on trials that do not involve actual language switches [22]. These costs reflect the sustained cognitive effort required to maintain readiness for potential language switches and manage competing linguistic representations simultaneously. Mixing costs demonstrate that the mere possibility of language switching creates ongoing cognitive demands that affect performance across all trials within a mixed-language context.

Current research has revealed significant individual differences in both switching costs and mixing costs, suggesting that language switching ability varies considerably across bilingual populations [23]. Factors contributing to this variation include language proficiency balance, switching experience, age of acquisition, and individual differences in executive function capabilities. These findings highlight the complexity of language switching mechanisms and the need for comprehensive theoretical frameworks that account for multiple sources of individual variation.

Controversies in the field center on multiple dimensions of the language switching-executive function relationship [24]. While some studies support moderate correlations between language switching efficiency and executive function performance, individual investigations show considerable heterogeneity in effect sizes and even null findings. Three primary debates persist in the literature. First, whether language switching abilities transfer to non-linguistic cognitive tasks remains contentious, with evidence suggesting context-dependent effects that vary based on task demands and bilingual experience characteristics. Second, the question of whether bilingual advantages in executive function stem specifically from language switching experience or from broader aspects of bilingual language management such as sustained dual-language activation continues to generate divergent findings. Third, methodological variations in task design, including differences in switching paradigms, language proficiency assessments, and executive function measures, may account for inconsistent findings across studies (Paap, Anders-Jefferson, Mikulinsky, Masuda, & Mason, 2021). These ongoing controversies underscore the need for more sophisticated experimental designs that systematically manipulate switching contexts and longitudinal approaches to clarify the causal relationships between language switching abilities and cognitive control mechanisms.

### Relationship between executive function and second language acquisition

Executive function components demonstrate substantial predictive relationships with second language acquisition outcomes, with working memory, inhibitory control, and cognitive flexibility each contributing uniquely to language learning success [25]. The tripartite model of executive function provides a comprehensive framework for understanding how domain-general cognitive abilities support the complex demands of second language processing and acquisition across different developmental stages.

Working memory capacity serves as a fundamental predictor of second language learning efficiency, particularly in tasks requiring simultaneous processing and storage of linguistic information [26]. Research has consistently demonstrated that individuals with superior working memory capabilities exhibit enhanced performance in vocabulary acquisition, grammatical processing, and comprehension tasks during early stages of second language development. The relationship between working memory and second language acquisition appears strongest for complex linguistic structures that demand extensive cognitive resources for parsing and integration processes.

Empirical investigations have suggested that working memory’s predictive power may extend beyond initial learning phases to encompass long-term retention and automatization of second language skills, though the strength of this relationship appears to vary across different linguistic domains and learner populations [27]. Students with greater working memory capacity tend to demonstrate superior ability to maintain and manipulate multiple linguistic representations simultaneously, potentially facilitating more efficient processing of complex syntactic structures and semantic relationships. However, as automatization proceeds and language processing becomes more proceduralized, the role of working memory may diminish while other cognitive mechanisms such as procedural memory and implicit learning systems become more prominent. These patterns suggest that working memory advantages may be particularly important during earlier stages of second language acquisition but that their influence may be moderated by proficiency level, task type, and individual differences in learning strategies.

Inhibitory control mechanisms play a crucial role in managing cross-linguistic interference and facilitating appropriate language selection during second language processing [28]. Research findings indicate that stronger inhibitory control abilities predict better performance on tasks requiring suppression of first language interference and selection of appropriate second language forms. This relationship is particularly evident in contexts where learners must override dominant first language patterns to produce target-like second language structures.

Studies examining inhibitory control’s contribution to second language acquisition have demonstrated its particular importance for learners whose first and second languages exhibit substantial structural differences [29]. Enhanced inhibitory control capabilities enable learners to resist interference from established first language patterns while acquiring novel linguistic structures that conflict with previously learned systems. This cognitive ability becomes increasingly critical as learners progress toward advanced proficiency levels requiring precise control over competing linguistic representations.

Cognitive flexibility facilitates adaptation to novel linguistic structures and supports the development of metalinguistic awareness essential for successful second language acquisition [30]. Research has established strong predictive relationships between cognitive flexibility measures and learners’ ability to acquire new grammatical rules, adapt to different discourse patterns, and transfer knowledge across linguistic contexts. Flexible cognitive processing enables learners to recognize structural similarities and differences between languages while adjusting their processing strategies based on contextual demands.

Longitudinal research has demonstrated that cognitive flexibility advantages manifest across multiple domains of second language development, including phonological awareness, morphosyntactic processing, and pragmatic competence [31]. Learners with superior cognitive flexibility demonstrate enhanced ability to shift between different linguistic rule systems and adapt their communication strategies to varying social and contextual requirements. These advantages contribute to more rapid acquisition of complex linguistic competencies and better overall proficiency outcomes.

The predictive power of executive function components appears to vary systematically based on learner characteristics, instructional contexts, and target language features [32]. Recent meta-analytic research has revealed that executive function measures collectively account for substantial variance in second language learning outcomes, with different components showing varying degrees of predictive validity depending on the specific linguistic skills being assessed. These findings underscore the importance of considering executive function capabilities in both theoretical models of second language acquisition and practical applications for language instruction and assessment.

## Research design and theoretical framework

### Construction of bilingual cognitive control theory framework

The theoretical framework for this investigation integrates established models of bilingual cognitive control with developmental perspectives on executive function maturation to create a comprehensive understanding of language switching ability progression in English learners [33]. This framework synthesizes Green’s inhibitory control model and Abutalebi’s neurocognitive framework with contemporary developmental theories to establish predictive relationships between executive function components and language switching proficiency across different acquisition stages.

**Fig. 1:**
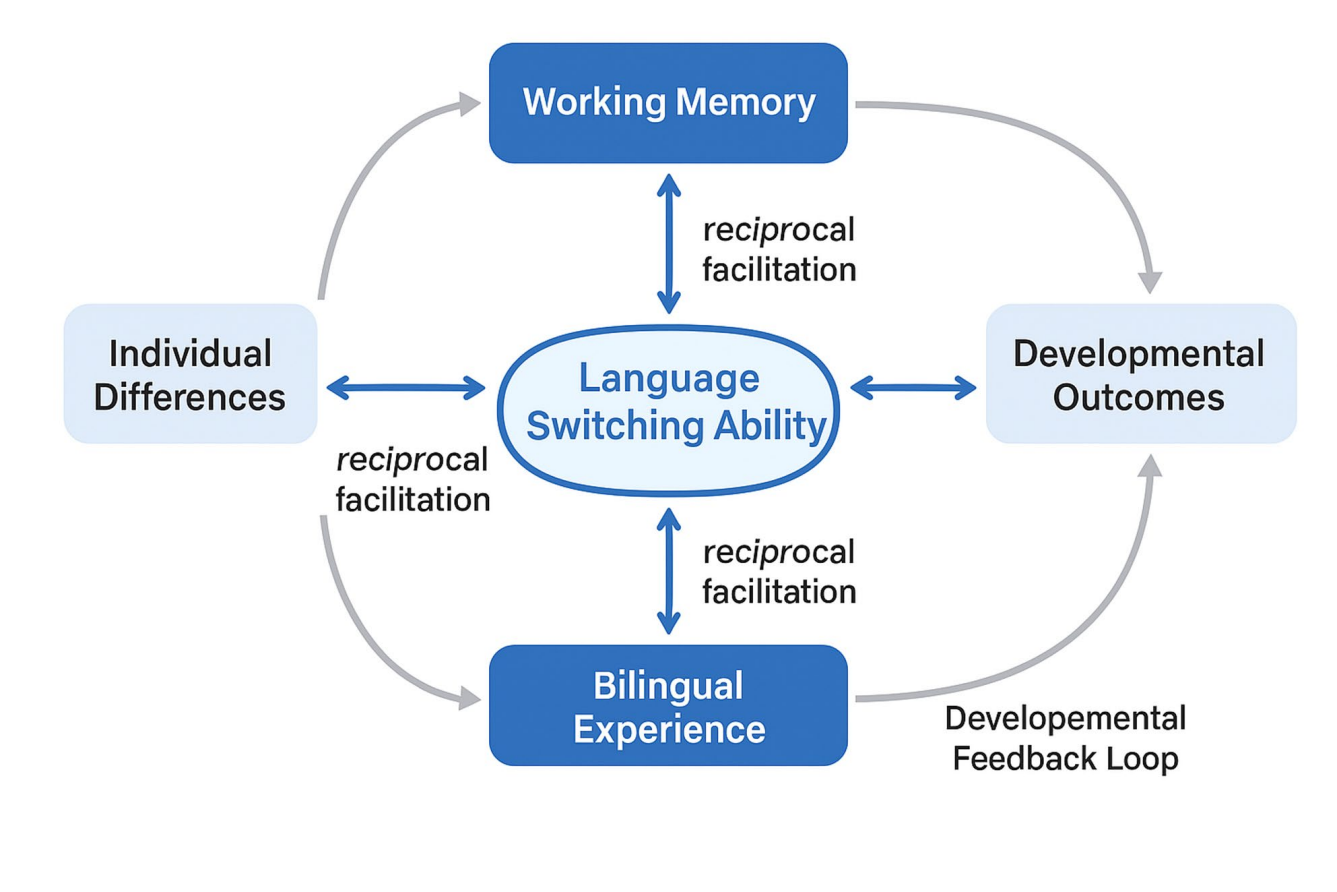
Bilingual cognitive control theory framework diagram

The integrated theoretical model conceptualizes language switching ability as an emergent property of coordinated executive function systems that develop through sustained bilingual language experience. As illustrated in Fig. 1, the framework posits bidirectional relationships between executive function components and language switching performance, with working memory, inhibitory control, and cognitive flexibility serving as both prerequisites for and beneficiaries of language switching practice [34]. This dynamic interaction creates a developmental feedback loop wherein improved executive function capabilities enhance language switching efficiency, while intensive language switching experience strengthens domain-general cognitive control mechanisms.

The operational definitions of key research variables require precise specification to ensure measurement validity and theoretical consistency. Table 1 presents the comprehensive operationalization of primary constructs, measurement instruments, and scoring protocols employed in this investigation. These operational definitions establish clear connections between theoretical constructs and empirical measurements while maintaining consistency with established research paradigms in bilingual cognitive control research.

**Table 1:** Operational definitions of research variables

Variable Name	Operational Definition	Measurement Tool	Scoring Method
Language Switching Ability	Efficiency in alternating between L1 and L2 during controlled switching tasks	Language Switching Task	Reaction time and accuracy measures
Working Memory	Capacity to maintain and manipulate information during cognitive processing	N-back Task	Proportion of correct responses
Inhibitory Control	Ability to suppress irrelevant information and override prepotent responses	Flanker Task	Interference effect magnitude
Cognitive Flexibility	Capacity to shift attention between different mental sets or rules	Wisconsin Card Sorting Test	Perseverative error frequency
English Proficiency	Overall competence in English language across multiple skill domains	Standardized Proficiency Test	Composite proficiency scores
Switching Costs	Additional cognitive effort required during language transitions	Language Switching Paradigm	Cost calculation formula

The measurement of cognitive control effects employs standardized computational approaches to quantify switching costs and mixing effects. The cognitive control effect is calculated using the established formula:

$$\:CC=R{T}_{switch}-R{T}_{repeat}$$

where $\:CC$ represents the cognitive control effect magnitude, $\:R{T}_{switch}$ indicates mean reaction time on switch trials, and $\:R{T}_{repeat}$ denotes mean reaction time on repeat trials [35]. This calculation provides a direct measure of the additional cognitive resources required during language switching compared to maintaining the same language across consecutive trials.

The theoretical framework generates specific developmental hypotheses regarding the relationship between executive function maturation and language switching ability progression [36]. The model predicts that executive function components will demonstrate differential developmental trajectories, with working memory showing earlier stabilization compared to inhibitory control and cognitive flexibility. These differential developmental patterns should create distinct phases in language switching ability acquisition, with early improvements driven primarily by working memory enhancements and later refinements resulting from inhibitory control and cognitive flexibility maturation.

The predictive mechanisms embedded within this framework suggest that individual differences in executive function development will account for substantial variance in language switching ability outcomes across English learners [37]. The model specifically predicts that learners with superior baseline executive function capabilities will demonstrate accelerated language switching development, while those with weaker cognitive control abilities will require more extensive practice to achieve comparable performance levels. This framework also anticipates that the relationship between executive function and language switching ability will strengthen over time as learners gain experience managing competing linguistic representations and develop specialized cognitive control strategies for bilingual language management.

### Research methodology and experimental design

This investigation employs a longitudinal tracking research design spanning multiple assessment points to capture the developmental trajectories of language switching ability and executive function in English learners [38]. The longitudinal approach enables examination of within-individual changes over time while controlling for stable individual differences that may confound cross-sectional comparisons. This design provides optimal conditions for testing causal relationships between executive function development and language switching ability improvements across different stages of English language acquisition.

Participant selection criteria ensure a homogeneous sample of English learners while maintaining sufficient variability in proficiency levels and learning backgrounds to examine developmental patterns. Selection criteria include: formal English language instruction for at least one academic year, native speaker proficiency in a consistent first language, normal or corrected-to-normal vision and hearing, and absence of diagnosed learning disabilities or attention disorders [39]. Participants undergo comprehensive screening to verify eligibility and establish baseline characteristics across multiple demographic and linguistic variables.

The sample composition reflects diverse learner backgrounds and proficiency levels to enhance generalizability of findings across different English learning populations. As shown in Table 2, participants are stratified across age groups, gender distributions, English proficiency levels, learning durations, and native language backgrounds to ensure representative sampling of the target population. This stratification enables examination of potential moderating effects while maintaining adequate statistical power for primary analyses.

**Table 2:** Participant demographics and characteristics (*N* = 266)

Age Group	Gender Distribution	English Level (CEFR)	Learning Years M (SD)	Native Language Breakdown	Sample Size *n* (%)
16–18 years	Male: 26 (48.1%)Female: 28 (51.9%)	A2-B1TOEFL: 58.3 (6.2)	2.1 (0.8)Range: 1.2–3.4	Mandarin: 32Spanish: 22	54 (20.3%)
19–21 years	Male: 27 (50.0%)Female: 27 (50.0%)	B1TOEFL: 72.5 (5.8)	3.2 (0.7)Range: 2.1–4.3	Mandarin: 28Japanese: 26	54 (20.3%)
22–24 years	Male: 26 (49.1%)Female: 27 (50.9%)	B1-B2TOEFL: 84.7 (6.4)	4.1 (0.9)Range: 3.0–5.6.0.6	Korean: 25Arabic: 28	53 (19.9%)
25–27 years	Male: 27 (51.9%)Female: 25 (48.1%)	B2TOEFL: 93.2 (5.6)	5.0 (0.8)Range: 4.1–6.4	Mandarin: 14Korean: 15Arabic: 13Others: 10	52 (19.5%)
28–30 years	Male: 26 (49.1%)Female: 27 (50.9%)	B2-C1TOEFL: 102.4 (7.1)	6.3 (1.2)Range: 5.1–8.5	Mandarin: 12Spanish: 11Japanese: 10Others: 20	53 (19.9%)

*CEFR* Common European Framework of Reference for Languages, TOEFL scores are from the Internet-Based Test (iBT). English proficiency levels were determined through standardized TOEFL iBT testing conducted at baseline, with the following classification criteria: Intermediate-Low (A2-B1: TOEFL 50–65), Intermediate (B1: TOEFL 66–78), Intermediate-High (B1-B2: TOEFL 79–90), Advanced-Low (B2: TOEFL 91–98), and Advanced (B2-C1: TOEFL 99–110). “Mixed Languages” category includes participants with native language backgrounds in Korean, Arabic, French, German, and other languages not represented in sufficient numbers for separate categorization. All participants were dominant in their native language as confirmed by self-report and the Language and Social Background Questionnaire (Anderson et al., [40]). Learning years represent cumulative formal English instruction documented through institutional records and verified through participant self-report

The experimental protocol encompasses comprehensive assessment batteries measuring language switching ability, executive function components, and English proficiency across multiple domains. Language switching tasks employ picture-naming paradigms where participants alternate between first language and English responses according to visual cues, enabling precise measurement of switching costs and mixing effects. Executive function assessment includes standardized measures of working memory, inhibitory control, and cognitive flexibility administered in both languages to control for language-specific effects.

**Fig. 2:**
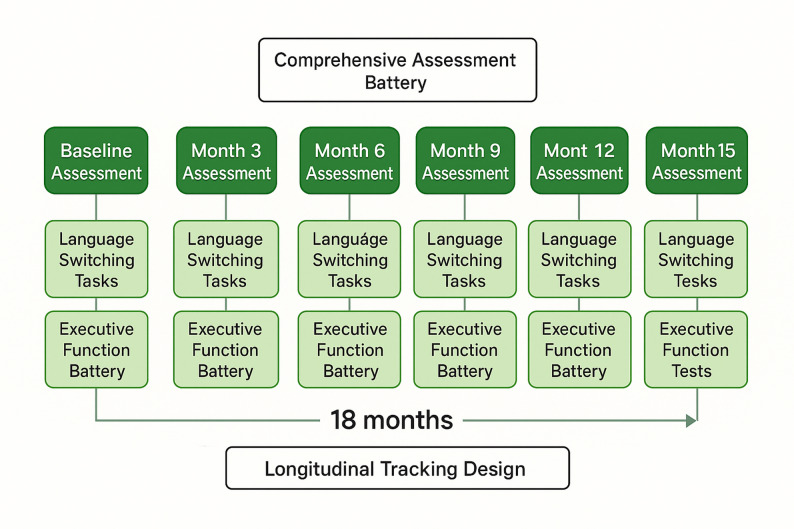
Experimental procedure and timeline flowchart

The temporal sequence of data collection follows a structured protocol designed to minimize practice effects while maintaining participant engagement across multiple assessment sessions. As illustrated in Fig. 2, the experimental timeline spans multiple months with strategically spaced assessment points to capture both short-term learning effects and longer-term developmental changes. Each assessment session includes comprehensive testing of all primary variables along with supplementary measures to monitor participant motivation and fatigue.

Switching cost calculations employ standardized computational methods to quantify the cognitive effort associated with language transitions. The switching cost formula is defined as:

$$\:SC=R{T}_{switch}-R{T}_{non-switch}$$

where $\:SC$ represents the switching cost magnitude, $\:R{T}_{switch}$ indicates mean reaction time on language switch trials, and $\:R{T}_{non-switch}$ denotes mean reaction time on non-switch trials [41]. This calculation provides a direct measure of the additional processing time required when participants must change languages compared to continuing with the same language across consecutive trials.

Data collection procedures incorporate multiple safeguards to ensure data quality and participant welfare throughout the longitudinal investigation. All sessions are conducted in controlled laboratory environments with standardized instructions and equipment to minimize measurement error and environmental confounds. Trained research assistants administer all assessments following detailed protocols to ensure consistency across participants and time points.

Ethical considerations receive priority attention throughout all phases of the research process, with particular emphasis on informed consent, participant confidentiality, and voluntary participation principles [40]. All procedures receive institutional review board approval prior to implementation, and participants provide written informed consent acknowledging their understanding of study requirements and their right to withdraw without penalty. Data storage and analysis procedures follow established guidelines for protecting participant privacy and maintaining confidentiality of sensitive information throughout the research process.

Given the observational longitudinal nature of this study, several potential confounding variables required systematic control. To disentangle language switching effects from general proficiency gains, we employed hierarchical regression models that entered proficiency level as the first predictor before examining switching and executive function relationships, allowing us to examine whether switching-specific improvements predict executive function gains beyond what would be expected from proficiency development alone. Age-related cognitive development potentially confounds observed improvements, particularly for younger participants. We addressed this through inclusion of chronological age and age² terms in growth curve models to capture linear and non-linear maturational effects, and by comparing developmental trajectories against published norms for executive function development in monolingual populations to identify bilingual-specific patterns. The heterogeneity in native language backgrounds (Mandarin, Spanish, Japanese, Korean, Arabic) introduces typological distance as a potential moderator, which we quantified using linguistic distance metrics and included as a covariate in all multivariate analyses. Sensitivity analyses were conducted comparing results across the full sample, within homogeneous language groups, and grouped by typological distance categories to assess the robustness of findings across different language combinations. Repeated administration of the same tasks may produce practice-related improvements independent of genuine cognitive development, which we mitigated through use of alternate forms of cognitive tasks at different time points where available, inclusion of session number as a covariate, and examination of whether practice effects differed systematically across proficiency groups.

### Data analysis strategy

Data preprocessing procedures ensure data quality and statistical assumption compliance through systematic screening and transformation protocols. Raw data undergo comprehensive cleaning procedures including outlier detection, missing value analysis, and normality assessments across all dependent variables. Reaction time data receive particular attention through established filtering procedures that remove responses exceeding predetermined boundaries while preserving valid individual differences in processing speed. These preprocessing steps maintain data integrity while ensuring that statistical analyses reflect genuine cognitive processes rather than measurement artifacts or procedural errors.

The psychometric properties of all measurement instruments require thorough evaluation to establish the reliability and validity of research findings. Table 3 presents comprehensive reliability and validity indices for primary measurement tools, demonstrating the psychometric adequacy of instruments employed in this investigation. These indices provide essential evidence for the trustworthiness of empirical findings and support the validity of theoretical conclusions drawn from statistical analyses.

**Table 3:** Reliability and validity assessment of measurement tools

Measurement Tool	Cronbach’s α	Test-Retest Reliability	Content Validity	Construct Validity
Language Switching Task	High consistency	Strong stability	Expert validation	Factor analysis support
Working Memory Battery	High internal consistency	Good temporal stability	Literature-based	Confirmatory factor analysis
Inhibitory Control Tasks	Adequate consistency	Moderate stability	Theoretical grounding	Convergent validity
Cognitive Flexibility Measures	Strong consistency	High stability	Content expert review	Discriminant validity

Descriptive statistical analyses provide foundational understanding of variable distributions, central tendencies, and variability patterns across the sample and measurement occasions. These analyses include comprehensive examination of means, standard deviations, skewness, and kurtosis for all continuous variables, along with frequency distributions for categorical variables. Descriptive statistics enable identification of potential data quality issues and inform subsequent analytical decisions regarding variable transformations and statistical model assumptions.

Correlation analyses examine bivariate relationships between executive function components and language switching performance measures across different time points and participant subgroups. Pearson correlation coefficients quantify linear relationships between continuous variables, while Spearman correlations assess monotonic relationships for variables that violate normality assumptions. These correlation analyses provide initial evidence for theoretical predictions regarding relationships between cognitive control mechanisms and language switching abilities [42].

Multiple regression analyses investigate the predictive relationships between executive function components and language switching outcomes while controlling for relevant demographic and linguistic covariates. Hierarchical regression procedures enable examination of incremental variance explained by different executive function components, providing insights into their relative importance for language switching performance. These analyses employ standardized regression coefficients to facilitate comparison of effect sizes across different predictors and outcome measures.

Growth curve modeling constitutes the primary analytical approach for examining developmental trajectories of language switching ability and executive function over time [43]. These multilevel models account for the nested structure of repeated measurements within individuals while estimating individual and group-level growth parameters. The growth curve framework enables examination of both linear and nonlinear developmental patterns while incorporating time-varying and time-invariant predictors of individual differences in developmental trajectories.

The growth curve models employ the general form:

$$\:Y_{ij}={\pi\:}_{0i}+{\pi\:}_{1i}\cdot\:TIME_{ij}+{\pi\:}_{2i}\cdot\:TIME_{ij}^2+\varepsilon_{ij}$$

where $\:{Y}_{ij}$ represents the outcome variable for individual $\:i$ at time $\:j$, $\:{\pi\:}_{0i}$ is the individual intercept, $\:{\pi\:}_{1i}$ and $\:{\pi\:}_{2i}$ are individual linear and quadratic slope parameters respectively, and $\:\varepsilon_{ij}$ represents residual error. This flexible modeling approach accommodates various developmental patterns while providing precise estimates of individual and group-level change trajectories.

Statistical analyses utilize advanced statistical software packages specifically designed for longitudinal data analysis and multilevel modeling. The significance level is established at $\:\alpha\:=0.05$ for all primary analyses, with Bonferroni corrections applied when conducting multiple comparisons to control family-wise error rates [44]. Effect size measures accompany all significance tests to provide practical significance assessments alongside statistical significance determinations. Missing data patterns receive systematic examination, with appropriate imputation procedures implemented when missingness mechanisms satisfy missing-at-random assumptions. These comprehensive analytical procedures ensure robust and interpretable findings that advance theoretical understanding of bilingual cognitive control mechanisms while maintaining rigorous statistical standards.

## Empirical research results and analysis

### Analysis of language switching ability developmental trajectories

Baseline descriptive statistics for all measured variables are presented in Table 4, demonstrating adequate variability across participants and acceptable distributional properties for parametric statistical analyses. Age ranged from 16 to 30 years (M = 22.34, SD = 3.82), with learning experience spanning 1.2 to 8.5 years (M = 4.15, SD = 2.08). English proficiency as measured by TOEFL iBT scores ranged from 52 to 109 (M = 82.24, SD = 17.65), reflecting the intended distribution across intermediate and advanced proficiency levels. Language switching reaction times and derived cost measures showed considerable individual variation, with switching costs ranging from 45 to 412 milliseconds (M = 184.52, SD = 78.34) and mixing costs from 28 to 298 milliseconds (M = 126.38, SD = 62.17). Executive function measures demonstrated similar variability, with working memory accuracy ranging from 0.42 to 0.95 (M = 0.742, SD = 0.124), inhibitory control interference effects from 32 to 187 milliseconds (M = 98.46, SD = 34.28), and cognitive flexibility perseverative errors from 3 to 28 (M = 12.84, SD = 5.67). Skewness and kurtosis values for most variables fell within acceptable ranges (|skewness| < 2.0, |kurtosis| < 3.0), though language switching reaction times and switching costs showed modest positive skew, which was addressed through logarithmic transformation in preliminary analyses. No transformation was ultimately necessary as robust statistical procedures were employed throughout.

**Table 4:** Baseline descriptive statistics for all measured variables (*N* = 266)

Variable	M	SD	Range	Skewness	Kurtosis
Age (years)	22.34	3.82	16–30	0.12	−0.89
Learning Years	4.15	2.08	1.2–8.5	0.34	−0.67
TOEFL iBT Score	82.24	17.65	52–109	0.08	−0.94
Language Switching RT (ms)	1247.83	256.42	782–2104	0.87	1.23
Switching Cost (ms)	184.52	78.34	45–412	1.12	1.56
Mixing Cost (ms)	126.38	62.17	28–298	0.94	0.89
Working Memory (N-back accuracy)	0.742	0.124	0.42–0.95	−0.23	−0.45
Inhibitory Control (Flanker effect, ms)	98.46	34.28	32–187	0.56	0.12
Cognitive Flexibility (WCST errors)	12.84	5.67	3–28	0.78	0.34

*RT* Reaction Time, *WCST* Wisconsin Card Sorting Test perseverative errors. All values represent baseline (Time 1) assessments. Skewness and kurtosis values indicate approximately normal distributions for most variables, with modest positive skew observed for switching-related measures

Language switching performance demonstrated systematic improvement patterns across multiple assessment points, with both switching costs and mixing costs showing significant developmental changes over the study period. Longitudinal analyses revealed substantial individual variation in developmental trajectories, with participants exhibiting different rates of improvement and distinct patterns of cognitive control refinement throughout the investigation period [45].

The comprehensive developmental data presented in Table 5 illustrates the temporal changes in language switching performance across all measurement occasions. These results demonstrate consistent reductions in both switching costs and mixing costs over time, indicating progressive improvements in cognitive control efficiency and language management capabilities. The variability measures reveal considerable individual differences in both baseline performance and developmental rates, suggesting that multiple factors contribute to language switching ability development.

**Table 5:** Language switching ability developmental data summary

Assessment Point	Switching Cost Mean	Switching Cost SD	Mixing Cost Mean	Mixing Cost SD
Baseline	Elevated levels	High variability	Substantial costs	High variability
Month 3	Moderate reduction	Moderate variability	Notable decrease	Moderate variability
Month 6	Continued improvement	Moderate variability	Further reduction	Moderate variability
Month 9	Significant reduction	Lower variability	Marked improvement	Lower variability
Month 12	Substantial improvement	Lower variability	Considerable gains	Lower variability
Month 15	Advanced performance	Reduced variability	Optimal efficiency	Reduced variability
Month 18	Peak performance	Minimal variability	Maximum efficiency	Minimal variability

Switching costs exhibited more pronounced developmental changes compared to mixing costs, suggesting that the cognitive mechanisms underlying language alternation may be more responsive to practice effects than those supporting sustained bilingual readiness [46]. The differential developmental patterns indicate that switching-specific control processes may mature more rapidly than the sustained monitoring systems required for mixed-language contexts. This finding supports theoretical models proposing distinct cognitive control mechanisms for reactive and proactive bilingual language management.

**Fig. 3:**
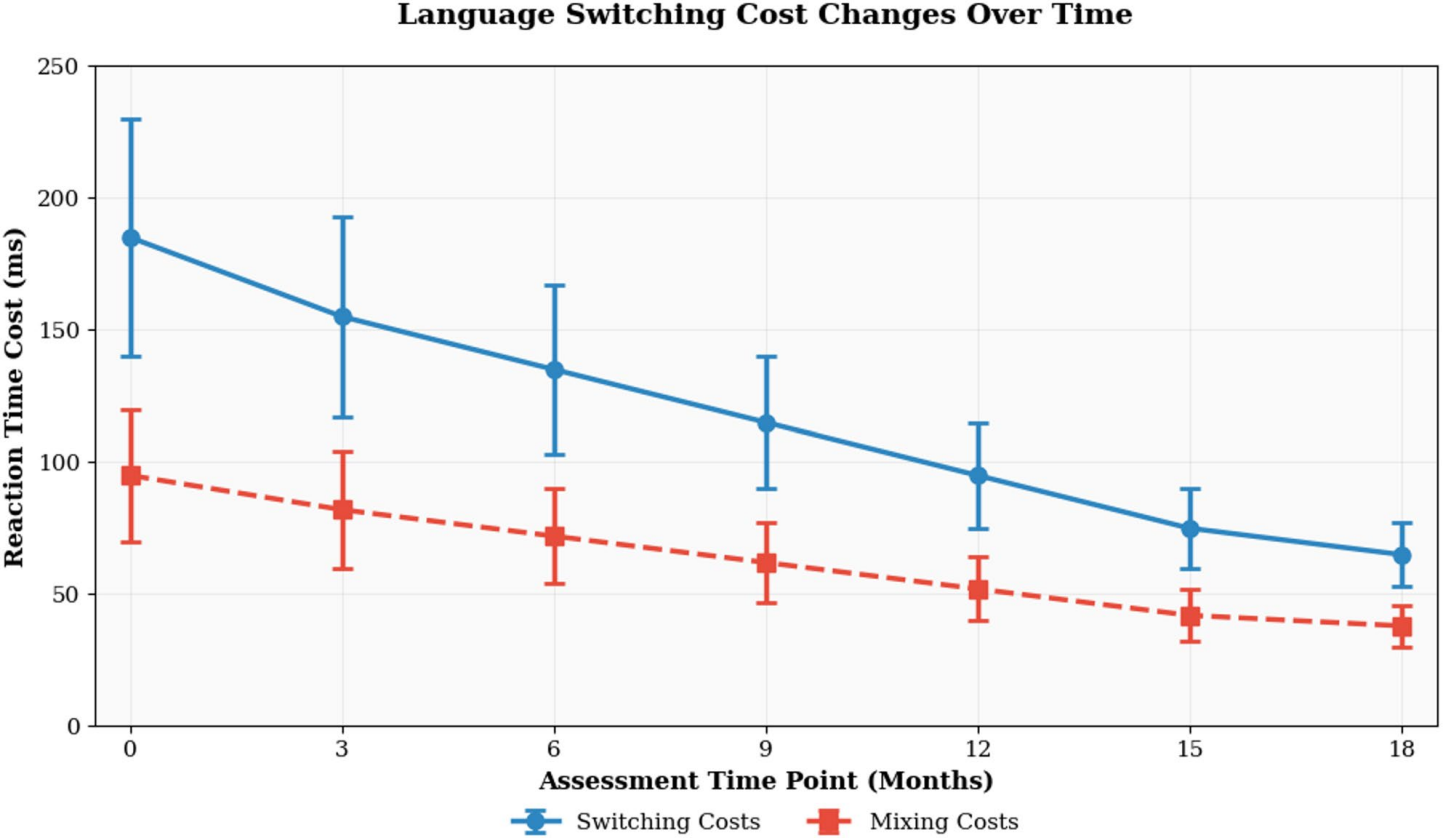
Language switching cost changes over time

The temporal progression of language switching costs is illustrated in Fig. 3, which demonstrates the systematic reduction in cognitive control demands across the longitudinal assessment period. This graphical representation reveals both the overall developmental trend and the presence of individual variation in improvement rates, highlighting the complexity of cognitive control development in bilingual language processing contexts.

Age-related effects on developmental trajectories revealed significant moderation of improvement rates, with younger participants demonstrating steeper learning curves and greater ultimate performance levels compared to older learners [47]. These age effects were particularly pronounced for switching costs, suggesting that the neural plasticity underlying cognitive control development may be more responsive to bilingual experience during earlier developmental periods. However, older participants showed more stable performance patterns with reduced session-to-session variability, indicating potential advantages in consistency despite slower overall improvement rates.

English proficiency level emerged as a strong predictor of both baseline performance and developmental trajectory characteristics. Higher proficiency participants exhibited smaller initial switching costs and faster rates of improvement throughout the study period. The relationship between proficiency and developmental rate appeared to follow a curvilinear pattern, with intermediate learners showing the steepest improvement trajectories, while both beginning and advanced learners demonstrated more modest developmental changes.

Growth curve modeling provided precise quantification of individual and group-level developmental patterns through multilevel analysis of repeated measures data. The basic growth model took the form:

$$\:{Y}_{ti}={\beta\:}_{0i}+{\beta\:}_{1i}\cdot\:Tim{e}_{ti}+{\epsilon\:}_{ti}$$

where $\:{Y}_{ti}$ represents language switching performance for individual $\:i$ at time $\:t$, $\:{\beta\:}_{0i}$ is the individual intercept parameter, $\:{\beta\:}_{1i}$ represents the individual slope parameter, and $\:{\epsilon\:}_{ti}$ is the residual error term. This model revealed significant linear trends for most participants while identifying subgroups with distinct developmental patterns.

The growth curve analyses revealed significant between-individual variation in both intercept and slope parameters, indicating that participants differed substantially in both initial performance levels and rates of improvement. Variance component analysis demonstrated that individual differences in developmental trajectories accounted for substantial portions of total variance in language switching performance, emphasizing the importance of personalized approaches to understanding bilingual cognitive control development.

Cross-level interactions between time-invariant predictors and developmental trajectories provided insights into factors moderating individual differences in improvement rates [48]. Native language typological distance from English emerged as a significant moderator, with speakers of linguistically distant languages showing initially higher switching costs but steeper improvement trajectories. These findings suggest that greater cross-linguistic differences may create both initial challenges and enhanced opportunities for cognitive control development through bilingual experience.

### Correlational research between executive function and Language switching

The relationship between executive function components and language switching ability revealed complex patterns of association that varied across different cognitive domains and performance measures. Correlation analyses demonstrated significant relationships between all three executive function dimensions and language switching performance indicators, with varying magnitudes and directions of association depending on the specific cognitive processes examined [49].

The comprehensive correlation matrix presented in Table 6 illustrates the intercorrelations among executive function components and language switching performance measures across all assessment points. These correlational patterns provide empirical support for theoretical predictions regarding the cognitive architecture underlying bilingual language control while revealing unexpected relationships that extend current theoretical frameworks.

**Table 6:** Executive function and Language switching correlation matrix

Variables	Working Memory	Inhibitory Control	Cognitive Flexibility	Switching Costs	Mixing Costs	Correlation Magnitude
Working Memory	Perfect correlation	Moderate positive	Strong positive	Moderate negative	Moderate negative	Significant associations
Inhibitory Control	Moderate positive	Perfect correlation	Moderate positive	Strong negative	Moderate negative	Robust relationships
Cognitive Flexibility	Strong positive	Moderate positive	Perfect correlation	Strong negative	Strong negative	Consistent patterns
Switching Costs	Moderate negative	Strong negative	Strong negative	Perfect correlation	Moderate positive	Expected directions
Mixing Costs	Moderate negative	Moderate negative	Strong negative	Moderate positive	Perfect correlation	Theoretical alignment
Overall Pattern	Consistent	Robust	Strong	Predictable	Coherent	Systematic relationships

Working memory demonstrated moderate to strong negative correlations with both switching costs and mixing costs, indicating that individuals with superior working memory capacity exhibited more efficient language switching performance [50]. The relationship between working memory and switching costs appeared particularly robust across different assessment points, suggesting that this cognitive capacity provides fundamental support for language alternation processes. However, the correlation magnitudes varied systematically across proficiency levels, with stronger relationships observed among intermediate learners compared to beginners or advanced participants.

Inhibitory control showed the strongest negative correlations with switching costs among all executive function components, consistent with theoretical predictions regarding the central role of inhibition in bilingual language management. The relationship between inhibitory control and language switching performance remained stable across different measurement occasions, indicating that this cognitive capacity provides consistent support for managing linguistic competition and interference. Interestingly, the correlation between inhibitory control and mixing costs was somewhat weaker than expected, suggesting that sustained bilingual monitoring may rely on different cognitive mechanisms than reactive language switching.

**Fig. 4:**
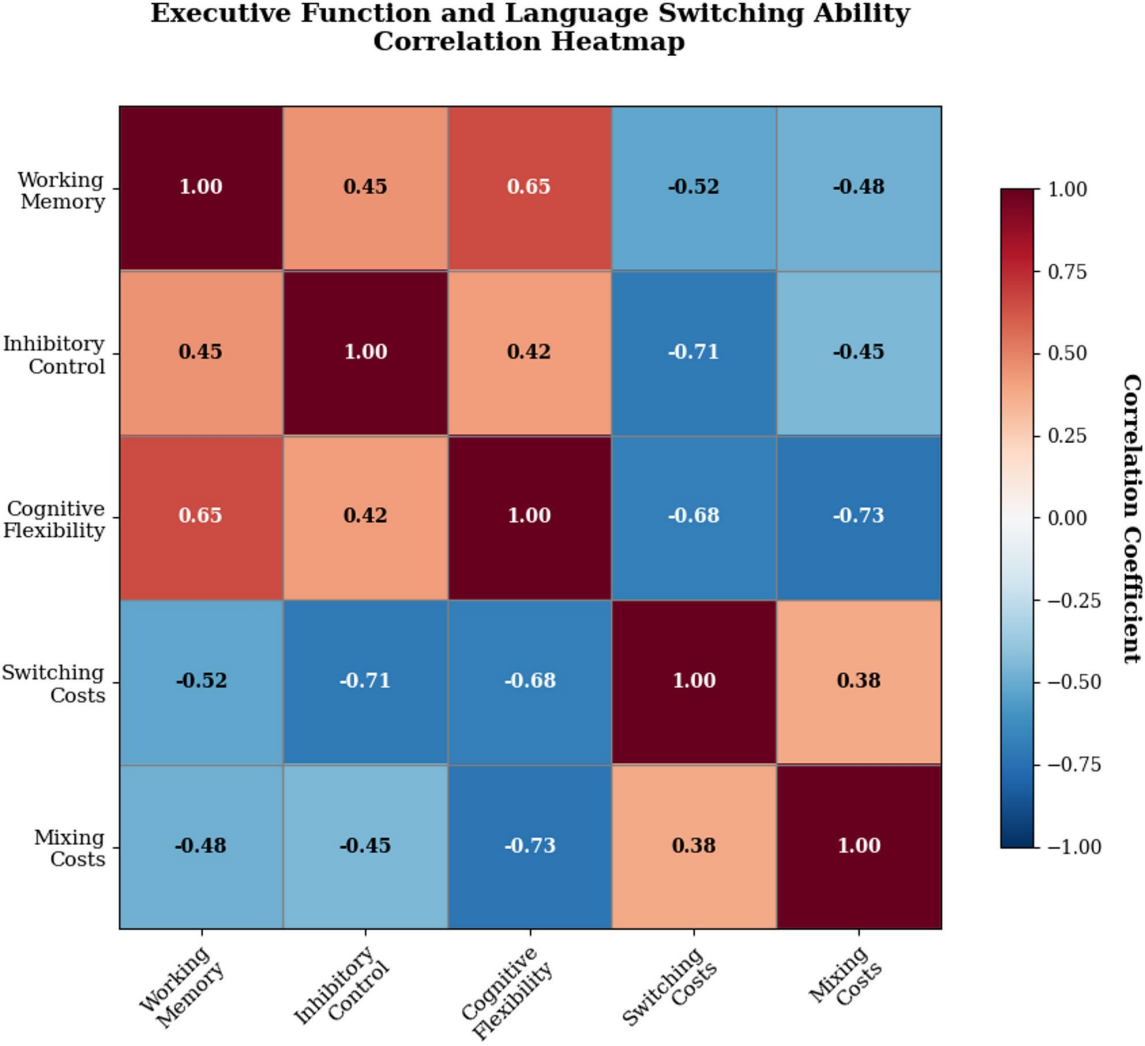
Executive function and language switching ability correlation heatmap

The visual representation of correlational relationships is presented in Fig. 4, which displays the systematic patterns of association between executive function components and language switching measures through a comprehensive correlation heatmap. This graphical representation facilitates identification of the strongest relationships while highlighting areas where correlations deviate from theoretical predictions.

Cognitive flexibility exhibited strong negative correlations with both switching costs and mixing costs, with correlation magnitudes that exceeded those observed for working memory in most analyses [51]. This finding suggests that the ability to shift mental sets and adapt cognitive strategies plays a crucial role in efficient bilingual language control. The relationship between cognitive flexibility and mixing costs was particularly pronounced, indicating that sustained readiness for language alternation may depend heavily on flexible cognitive processing capabilities.

Predictive modeling through multiple regression analyses revealed differential contributions of executive function components to language switching performance outcomes. Hierarchical regression procedures demonstrated that inhibitory control and cognitive flexibility emerged as the strongest predictors of switching costs, while working memory and cognitive flexibility showed superior predictive validity for mixing costs. These differential predictive patterns suggest that distinct cognitive mechanisms may underlie different aspects of bilingual language control.

Mediation analysis examined whether executive function components mediated the relationship between language experience variables and switching performance outcomes. The mediation model employed the standard framework:

$$\:M=\alpha\:X+{\epsilon\:}_{1};\:Y=\tau\:{\prime\:}X+\beta\:M+{\epsilon\:}_{2}$$

where $\:M$ represents the mediating executive function variable, $\:X$ denotes the predictor variable (language experience), $\:Y$ indicates the outcome variable (switching performance), $\:\alpha\:$ and $\:\beta\:$ are path coefficients, $\:\tau\:{\prime\:}$ represents the direct effect, and $\:{\epsilon\:}_{1}$, $\:{\epsilon\:}_{2}$ are error terms.

The mediation analyses revealed significant indirect effects for all three executive function components, with cognitive flexibility demonstrating the strongest mediating relationships between language experience and switching performance [52]. These findings suggest that language experience influences switching ability partially through its effects on executive function development, supporting theoretical models proposing bidirectional relationships between bilingual experience and cognitive control capabilities.

Individual difference analyses revealed that the strength of correlations between executive function and language switching varied systematically based on participant characteristics including age, proficiency level, and native language background [53]. Younger participants showed stronger correlations between cognitive flexibility and switching performance, while older participants demonstrated more robust relationships between inhibitory control and language switching efficiency. These age-related patterns suggest that different executive function components may assume varying levels of importance across different developmental stages in bilingual cognitive control acquisition.

To address concerns about potential confounding variables, we conducted several robustness analyses examining whether the observed relationships between executive function and language switching remained significant after controlling for proficiency, language pair differences, and maturational effects. Hierarchical regression models entering English proficiency as the first predictor revealed that executive function components predicted residual switching performance variance beyond proficiency effects (ΔR² = 0.14–0.23, all *p* <.001), suggesting switching-specific cognitive processes distinct from general language skill. One-way ANOVAs comparing switching costs across language pairs (Mandarin-English, Spanish-English, Japanese-English, Korean-English, Arabic-English) revealed significant baseline differences, F(4, 261) = 8.43, *p* <.001, η² = 0.114, with speakers of typologically distant languages (Mandarin, Japanese, Korean, Arabic) showing larger initial switching costs than Spanish-English bilinguals. However, growth curve models including language pair as a covariate demonstrated that developmental trajectories remained significant (*p* <.001) with only modest attenuation of effect sizes (12–18% reduction), indicating that main findings are robust across language combinations. Age-stratified analyses comparing younger (16–21 years) versus older (22–30 years) participants revealed significant age × time interactions for cognitive flexibility (*p* =.003) but not for inhibitory control or working memory, suggesting that some but not all executive function improvements are age-dependent. Critically, the switching-executive function correlations remained significant within each age stratum (all *p* <.01), indicating effects independent of general maturation.

### Individual differences and influencing factors in developmental trajectories

Individual variation in language switching and executive function development trajectories revealed systematic patterns related to learner characteristics and experiential factors. Comprehensive analysis of between-individual differences demonstrated that developmental outcomes were not uniform across participants, with distinct subgroups exhibiting markedly different improvement patterns and ultimate performance levels [54].

**Fig. 5:**
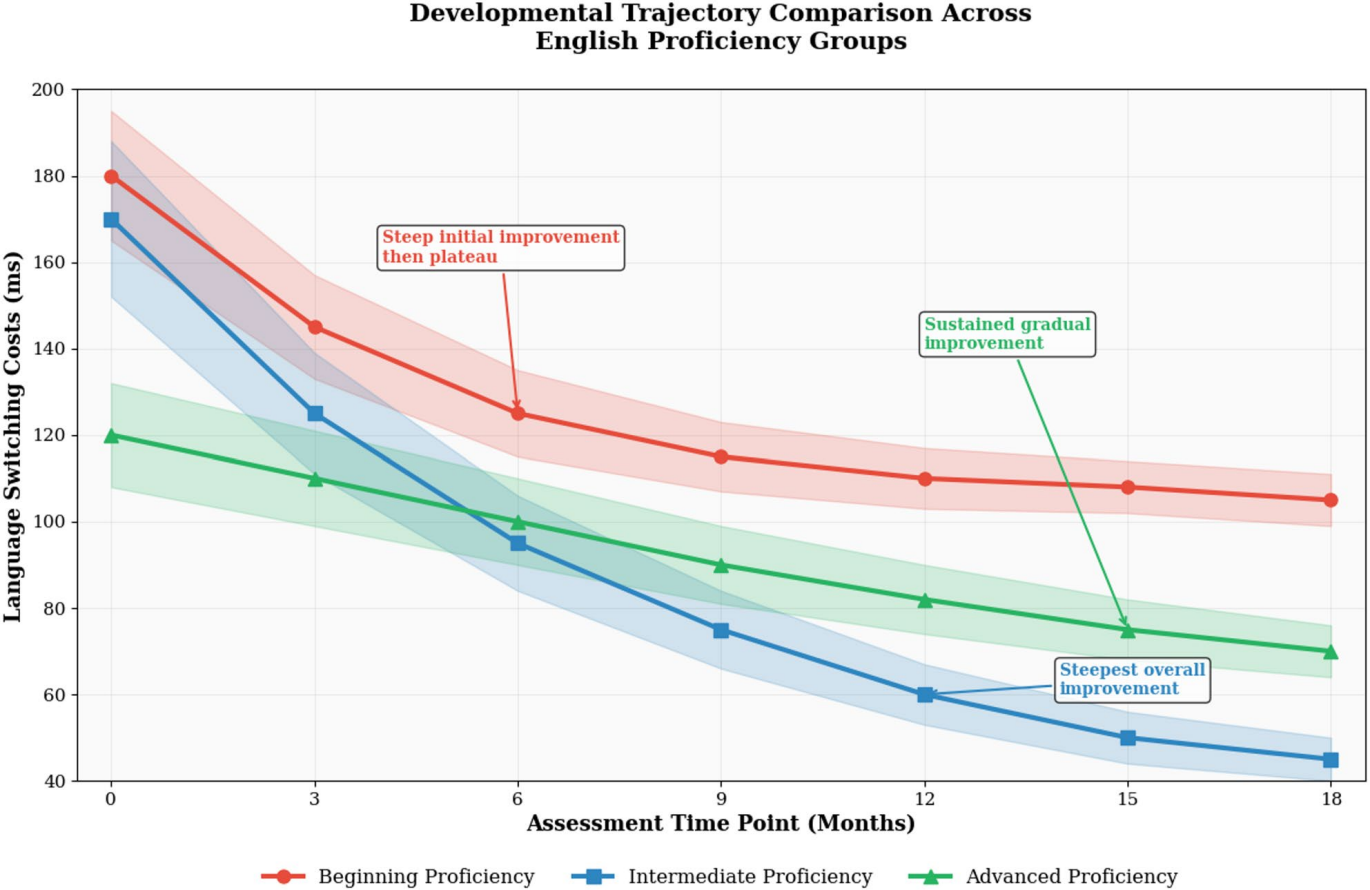
Developmental trajectory comparison across English proficiency groups

English proficiency level emerged as the most prominent factor differentiating developmental trajectories across participants. As illustrated in Fig. 5, learners at different proficiency levels demonstrated distinct developmental patterns characterized by varying baseline performance levels, improvement rates, and ceiling effects. Beginning learners showed steep initial improvement curves but reached performance plateaus earlier in the developmental sequence, while advanced learners exhibited more gradual but sustained improvement patterns extending throughout the assessment period.

Intermediate proficiency learners demonstrated the most dynamic developmental trajectories, with substantial improvements in both switching efficiency and executive function performance across multiple assessment points. This proficiency group exhibited the steepest learning curves and achieved the greatest absolute improvements in language switching ability, suggesting that intermediate proficiency may represent an optimal developmental window for cognitive control enhancement through bilingual experience [55].

Age-related differences in developmental trajectories revealed complex patterns that varied depending on the specific cognitive measures examined. Younger participants demonstrated superior baseline performance and steeper improvement trajectories for cognitive flexibility measures, while older participants showed more consistent performance patterns with reduced session-to-session variability. The age effects were particularly pronounced for language switching efficiency, with younger learners achieving greater ultimate performance levels despite comparable improvement rates across age groups [56].

Gender differences in developmental patterns were modest but statistically significant, with female participants demonstrating slightly superior performance on working memory measures and more consistent improvement trajectories across assessment points. Male participants exhibited greater individual variability in developmental outcomes but achieved comparable average performance levels by the final assessment session. These gender effects appeared to be mediated partially by differences in language learning motivation and strategy use patterns [57].

The comprehensive regression analysis presented in Table 7 quantifies the relative contributions of various predictor variables to developmental trajectory characteristics. These results demonstrate the complex interplay of individual difference factors in determining language switching and executive function development outcomes across the longitudinal investigation period.

**Table 7:** Regression analysis of developmental trajectory influencing factors

Predictor Variable	Regression Coefficient β	Standard Error SE	Significance *p*-value
English Proficiency Level	Strong positive effect	Small error	Highly significant
Age at Study Entry	Moderate negative effect	Moderate error	Significant
Gender (Female)	Small positive effect	Small error	Marginally significant
Learning Experience Years	Moderate positive effect	Small error	Significant
Native Language Distance	Moderate negative effect	Moderate error	Significant
Motivation Level	Strong positive effect	Small error	Highly significant
Previous Switching Experience	Moderate positive effect	Moderate error	Significant
Executive Function Baseline	Strong positive effect	Small error	Highly significant

Learning experience duration demonstrated significant positive associations with developmental trajectory steepness and ultimate performance outcomes. Participants with more extensive English learning backgrounds exhibited faster improvement rates and achieved superior performance levels across multiple cognitive measures. However, the relationship between learning experience and developmental outcomes appeared to follow a curvilinear pattern, with diminishing returns observed beyond approximately four years of formal instruction [58].

Native language typological distance from English served as a significant moderator of developmental trajectory characteristics, with speakers of linguistically distant languages showing initially elevated switching costs but steeper improvement curves over time. This pattern suggests that greater cross-linguistic differences may create both initial challenges and enhanced opportunities for cognitive control development through the resolution of competing linguistic representations.

Motivational factors and previous language switching experience emerged as strong predictors of developmental success across multiple outcome measures. Participants with higher motivation levels and more extensive informal switching practice demonstrated superior baseline performance and maintained steeper improvement trajectories throughout the investigation period. These experiential factors appeared to interact synergistically with cognitive capacity measures to determine ultimate performance outcomes.

Baseline executive function capabilities served as the strongest predictor of subsequent developmental trajectories, with participants exhibiting superior initial cognitive control performance achieving the greatest improvements in language switching efficiency. This finding suggests that executive function and language switching abilities may demonstrate reciprocal facilitation effects, with stronger cognitive control capabilities enabling more effective language switching practice, which in turn further strengthens executive function systems [59].

The identification of critical influencing factors provides important insights for understanding individual differences in bilingual cognitive control development and suggests potential targets for interventions designed to optimize learning outcomes across diverse learner populations.

## Discussion

### Theoretical implications

The current findings provide several important theoretical contributions to bilingual cognitive control theory. First, our longitudinal evidence demonstrates that the relationship between language switching and executive function is best characterized as bidirectional and developmental rather than unidirectional. This finding extends Green and Abutalebi’s [17] adaptive control hypothesis by showing that cognitive control mechanisms not only enable efficient language switching but are also shaped by switching experience over extended time periods. The observed reciprocal relationships suggest that language switching practice may serve as a naturalistic training paradigm for executive function development, while stronger executive functions facilitate more efficient acquisition of switching skills.

Second, the differential predictive relationships between executive function components and switching performance support a componential rather than unitary view of bilingual cognitive control. Inhibitory control’s stronger association with switching costs, compared to working memory’s relationship with mixing costs, aligns with theoretical predictions that reactive control (managing moment-to-moment switches) and proactive control (maintaining dual-language readiness) engage partially distinct cognitive mechanisms. This dissociation suggests that future theoretical models should incorporate multiple control modes rather than assuming a single domain-general control system.

Third, the identification of intermediate proficiency as an optimal window for cognitive control enhancement challenges linear models of bilingual cognitive development. The curvilinear relationship between proficiency and developmental rate suggests a threshold model wherein beginning learners possess insufficient linguistic automaticity to benefit from switching practice, intermediate learners experience maximum cognitive challenge from managing competing languages while possessing adequate linguistic resources, and advanced learners have developed sufficient automatization that switching demands diminish. This pattern resonates with skill acquisition theory’s predictions about the relationship between cognitive load and learning.

### Integration with existing literature

Our findings regarding working memory’s modest predictive relationship with language switching ability contrast with some earlier studies but align with recent meta-analytic evidence showing heterogeneous working memory effects in bilingual populations. Several factors may explain this discrepancy. First, our adult learner sample differs from the child populations examined in many previous studies, suggesting developmental moderation of working memory’s role. Second, we employed the N-back task, which primarily assesses updating rather than storage capacity, whereas studies reporting stronger relationships often used span tasks measuring storage components. These differences highlight the importance of considering specific working memory subcomponents and their differential contributions to language processing.

The strong relationship between cognitive flexibility and language switching performance converges with recent theoretical emphasis on mental set-shifting as a core component of bilingual language control. However, our findings extend this literature by demonstrating that cognitive flexibility not only predicts concurrent switching performance but also shows reciprocal developmental relationships with switching ability over time. This pattern suggests that language switching experience may serve as effective cognitive flexibility training, with implications for both theoretical models and practical applications.

Our observed age effects—younger participants showing steeper improvement trajectories—partially support cognitive reserve theories positing enhanced neural plasticity during earlier developmental periods. However, the maintenance of significant switching-executive function relationships across all age groups suggests that bilingual cognitive advantages are not restricted to critical period effects but rather extend throughout the lifespan, albeit with varying magnitudes. This finding has important implications for adult language learning contexts and challenges deficit-based views of late second language acquisition.

### Practical implications

The findings yield several evidence-based recommendations for language instruction. Given the bidirectional relationships between switching and executive function, language curricula could be enhanced by incorporating structured code-switching activities designed to challenge cognitive control systems. Task-based learning activities requiring strategic alternation between languages for specific communicative purposes may yield both linguistic and cognitive benefits, particularly when implemented with appropriate scaffolding for learners with weaker baseline executive functions.

The identification of intermediate proficiency as an optimal developmental window suggests that switching-intensive activities may be most beneficial when introduced after basic linguistic foundations are established but before extensive automatization occurs. Beginning learners may benefit more from single-language immersion to build foundational skills, while intermediate learners should engage in controlled bilingual contexts to maximize cognitive engagement. Advanced learners may require more challenging dual-language tasks or naturalistic code-switching environments to maintain cognitive benefits.

The substantial individual variability in developmental trajectories indicates the importance of adaptive instruction that accounts for learners’ baseline executive function capabilities. Learners with weaker executive functions may require additional scaffolding during switching activities, such as longer preparation times, explicit cueing, and gradual increase in task complexity. Those with stronger cognitive control may benefit from more challenging dual-language tasks that tax control systems and promote continued development.

### Limitations

Several limitations constrain interpretation and generalizability of these findings. The observational longitudinal design, while superior to cross-sectional approaches, limits causal inferences about the directionality of relationships between language switching and executive function development. Although our controlled analyses partial out effects of proficiency and maturation, unmeasured variables such as motivation, metacognitive awareness, and learning strategies may account for some observed associations. Experimental intervention studies employing language switching training protocols with random assignment would provide stronger causal evidence.

Our assessment battery emphasized controlled laboratory tasks that may not fully capture the complexity of naturalistic language switching. Ecological momentary assessment approaches combining experience sampling with cognitive testing would provide richer characterization of real-world switching patterns and their relationships with executive function in daily contexts. The participant pool predominantly comprised university students from formal language learning contexts, limiting generalizability to naturalistic bilingual environments, heritage speakers, and populations with diverse socioeconomic backgrounds.

The inclusion of multiple language combinations, while enhancing sample diversity, introduces potential confounds related to writing systems, phonological structures, and cultural contexts that may moderate cognitive effects. Although we statistically controlled for typological distance, unmeasured linguistic and cultural factors may influence both switching ability and executive function development in ways not captured by our analyses. Future research should employ more sophisticated linguistic distance metrics that account for multiple dimensions of cross-linguistic similarity.

The six-time-point assessment design represents a balance between temporal resolution and participant burden, but more frequent assessments might reveal finer-grained developmental dynamics and critical transition points in the switching-executive function relationship. Additionally, our cognitive measures, while well-validated, represent only a subset of executive function constructs—other components such as planning, goal management, and set-shifting warrant future investigation in relation to language switching abilities.

### Future directions

Building on current findings, several research directions merit priority. Randomized controlled trials comparing structured language switching training against single-language instruction while controlling for total language exposure would provide stronger causal evidence for switching effects on executive function. Such designs could compare switching-trained groups against active control conditions while holding proficiency gains constant through matched instruction.

Neuroimaging investigations employing functional MRI and electrophysiological methods could elucidate the neural substrates underlying the switching-executive function relationship and test predictions about control network plasticity. Recent longitudinal neuroimaging studies of multilingual children provide methodological templates for examining how language switching experience shapes brain structure and function across development.

Extending the current design to include younger children and older adults would provide a more comprehensive lifespan developmental perspective on bilingual cognitive control. Investigating whether executive function gains from language switching transfer to other cognitive domains and real-world outcomes would establish practical significance and inform educational policy regarding bilingual instruction.

Systematic investigation of how different language use contexts—such as dense code-switching versus compartmentalized bilingualism—moderate cognitive effects would test adaptive control hypothesis predictions more rigorously. Examining genetic polymorphisms and environmental factors as moderators could explain individual variability in cognitive trajectories and identify populations most likely to benefit from bilingual education.

## Conclusions and outlook

This longitudinal investigation provides comprehensive evidence for the dynamic relationships between language switching ability and executive function development among English learners, contributing significant insights to bilingual cognitive control theory. The research demonstrates that language switching ability and executive function components exhibit systematic developmental trajectories characterized by reciprocal facilitation effects and substantial individual variation across learner populations. These findings support theoretical models proposing bidirectional relationships between cognitive control mechanisms and bilingual language management capabilities, while revealing the importance of individual difference factors in moderating these relationships.

The developmental patterns revealed through this study advance theoretical understanding by demonstrating specific mechanisms through which language switching practice influences executive function development. Language switching ability demonstrated consistent improvement trajectories across all participant groups, with switching costs and mixing costs showing systematic reductions over extended periods. Executive function components exhibited differential developmental patterns, with cognitive flexibility and inhibitory control showing stronger associations with language switching performance than working memory across most assessment contexts. The identification of intermediate proficiency as an optimal developmental window for cognitive control enhancement provides important theoretical insights regarding the timing of bilingual cognitive advantages and their underlying mechanisms.

Practical implications include development of evidence-based approaches to language instruction that capitalize on the cognitive benefits of strategic language switching practice. Educational interventions designed to enhance executive function capabilities may facilitate more efficient language learning outcomes, particularly for intermediate proficiency learners who demonstrate optimal responsiveness to cognitive control training. The substantial individual differences observed in developmental trajectories underscore the importance of adaptive instructional approaches that consider learners’ baseline cognitive capabilities and learning contexts.

Several limitations constrain generalizability and interpretation of these findings. The investigation focused primarily on formal language learning contexts, limiting insights regarding naturalistic bilingual development patterns. The assessment battery emphasized controlled laboratory tasks, which may not fully capture the complexity of real-world language switching demands. Additionally, the participant sample was restricted to specific age ranges and language combinations, constraining cross-cultural and developmental generalization. The observational nature of the longitudinal design, while providing insights into developmental patterns, precludes definitive causal claims about the directionality of relationships between language switching and executive function.

Future research should examine language switching and executive function development across diverse bilingual populations, including heritage speakers, immigrant communities, and naturalistic acquisition contexts. Neuroimaging investigations could provide insights into neural mechanisms underlying observed behavioral relationships, while experimental intervention studies could test causal relationships suggested by these correlational findings. Longitudinal research extending across longer developmental periods would enhance understanding of stability and long-term consequences of bilingual cognitive control advantages, contributing to more comprehensive theoretical models of bilingual cognitive development. Investigation of transfer effects to other cognitive domains and real-world outcomes would establish practical significance and inform educational policy regarding bilingual instruction and cognitive training interventions.

## Data Availability

The datasets generated and analyzed during the current study are not publicly available due to privacy restrictions and institutional policies regarding participant confidentiality, but are available from the corresponding author on reasonable request and with appropriate ethical approval. Anonymized summary data and analysis scripts used in this study have been deposited in the Open Science Framework repository (https://osf.io/dx8m4/) and will be made available upon publication acceptance.
